# Beat alignment ability is associated with formal musical training not current music playing

**DOI:** 10.3389/fpsyg.2023.1034561

**Published:** 2023-01-30

**Authors:** Connor Spiech, Tor Endestad, Bruno Laeng, Anne Danielsen, E. F. Haghish

**Affiliations:** ^1^RITMO Centre for Interdisciplinary Studies in Rhythm, Time and Motion, University of Oslo, Oslo, Norway; ^2^Department of Psychology, University of Oslo, Oslo, Norway; ^3^Department of Musicology, University of Oslo, Oslo, Norway

**Keywords:** beat perception, active musicians, inactive musicians, nonmusicians, machine learning, musical training, beat alignment

## Abstract

The ability to perceive the beat in music is crucial for both music listeners and players with expert musicians being notably skilled at noticing fine deviations in the beat. However, it is unclear whether this beat perception ability is enhanced in trained musicians who continue to practice relative to musicians who no longer play. Thus, we investigated this by comparing active musicians’, inactive musicians’, and nonmusicians’ beat alignment ability scores on the Computerized Adaptive Beat Alignment Test (CA-BAT). 97 adults with diverse musical experience participated in the study, reporting their years of formal musical training, number of instruments played, hours of weekly music playing, and hours of weekly music listening, in addition to their demographic information. While initial tests between groups indicated active musicians outperformed inactive musicians and nonmusicians on the CA-BAT, a generalized linear regression analysis showed that there was no significant difference once differences in musical training had been accounted for. To ensure that our results were not impacted by multicollinearity between music-related variables, nonparametric and nonlinear machine learning regressions were employed and confirmed that years of formal musical training was the only significant predictor of beat alignment ability. These results suggest that expertly perceiving fine differences in the beat is not a use-dependent ability that degrades without regular maintenance through practice or musical engagement. Instead, better beat alignment appears to be associated with more musical training regardless of continued use.

## 1. Introduction

### 1.1. Background

Practice is said to make perfect, but do you remain perfect if you stop practicing or do you lose it if you do not use it? Humans’ ability to master a variety of skills has fascinated psychologists and neuroscientists for decades, culminating in a vast domain in and of itself (Ericsson and Charness, 1994; Sternberg and Grigorenko, 2003; Feltovich et al., 2006; Chi et al., 2014), spanning everything from sports (Shea and Paull, 2014) and dance (Calvo-Merino et al., 2005, 2006, 2010; Orgs et al., 2008, 2018) to beer and wine tasting (Tempere et al., 2019; Hinojosa-Aguayo et al., 2022). These impressive abilities are often described as the result of extensive practice and effort (Sloboda et al., 1996; Lehmann et al., 2018). Of particular interest to this work is how these factors impact musical expertise (Sloboda, 1991; Lehmann et al., 2018) and more importantly, whether they have a lasting effect once the practice comes to an end.

Musicians refine a number of different perceptual, motor, and cognitive skills to play their instrument(s) with fluency (Sloboda, 1991; Lehmann et al., 2007). Psychologically, this manifests in musicians outperforming nonmusicians in discriminating different pitches (Kishon-Rabin et al., 2001; Tervaniemi et al., 2005; Micheyl et al., 2006), tapping to rhythms (Franěk et al., 1991; Repp and Doggett, 2007; Chen et al., 2008; Skaansar et al., 2019), and remembering auditory stimuli (Pallesen et al., 2010; Cohen et al., 2011; Talamini et al., 2018). With the advent of neuroimaging tools, many studies have now shown that these differences manifest in a host of functional and anatomical changes to the brain as well (Amunts et al., 1997; Brattico et al., 2001; Imfeld et al., 2009; Jäncke, 2009; Dawson, 2014; Criscuolo et al., 2022). While there are clearly many investigations in the literature comparing musicians and nonmusicians, fewer break the dichotomy down into different types of musicianship and when they do, the results are often mixed. For instance, some studies have compared professional to amateur musicians and nonmusicians (Kauffman and Carlsen, 1989; Gaser and Schlaug, 2003; Hove et al., 2010; Krause et al., 2010; Repp, 2010; Mikutta et al., 2014; Appelgren et al., 2019), early- vs. late-trained musicians (Watanabe et al., 2007; Bailey and Penhune, 2010, 2013; Steele et al., 2013; Bailey et al., 2014; Shenker et al., 2022), and active vs. inactive musicians (Hanna-Pladdy and Gajewski, 2012; Bonde et al., 2018; Romeiser et al., 2021). This last classification of active vs. inactive musicians is especially important for investigating how ingrained these musical abilities truly are—do they dull without regular maintenance or are they set in stone once perfected?

One foundational musical ability is beat perception, the ability to detect temporal periodicities in musical rhythms (Schulze, 1978; Nguyen et al., 2018). The behavioral literature comparing beat perception in musicians and nonmusicians is somewhat mixed (Madsen, 1979; Rammsayer and Altenmüller, 2006; Grahn and Rowe, 2009; Mosing et al., 2014) with clear individual differences present (Grahn and McAuley, 2009; Grahn and Schuit, 2012). Specifically, musicians have been shown to be more accurate in judging tempo (Madsen, 1979) and beat alignment (Grahn and Schuit, 2012) as well as displaying better rhythm perception (Rammsayer and Altenmüller, 2006) and greater subjective experience of the beat, but only when one was present (Grahn and Rowe, 2009). Conversely, some of these researchers have found no difference between musicians and nonmusicians on similar rhythmic tasks like rhythm discrimination (Grahn and Brett, 2007), temporal generalization (Rammsayer and Altenmüller, 2006), beat strength tasks (after an outlier was removed; Grahn and McAuley, 2009), or musical aptitude in general once genetic influences were accounted for Mosing et al. (2014). This could be due to the multidimensionality of rhythmic abilities in general, with many different perceptual, cognitive, and genetic factors contributing (Mosing et al., 2014, 2016; Bartholomew et al., 2015; Hambrick and Tucker-Drob, 2015; Wesseldijk et al., 2019; Fiveash et al., 2022; Niarchou et al., 2022). However, another factor that could potentially distinguish differences in beat perception between musicians that has remained largely unexplored is whether rhythmic abilities change through continued music playing or devolve when discontinued.

### 1.2. The present study

Therefore, the present study was conducted to investigate possible differences in beat perception between active and inactive musicians and nonmusicians. We hypothesized that trained musicians who continue to play music regularly would be able to discriminate finer deviations from the beat (hereafter referred to as “beat alignment”) than nonmusicians and, to a lesser degree, inactive musicians who no longer play their instruments. A working hypothesis based on the “use it or lose it” principle of brain plasticity (e.g., Shors et al., 2012) further suggests that inactive musicians may simply revert to a previous stage of the ability, though it is unclear whether this stage is comparable to or more advanced than nonmusicians. Alternatively, sufficient musical training may cement heightened abilities regardless of regular rehearsal or other metrics of musical engagement. Conversely, greater musical engagement (reflected in regular playing) could be a result of greater beat alignment ability since those with lesser ability may be discouraged from continued playing.

As a follow-up control analysis, we also investigated the role of other demographics and musical engagement factors like years of formal musical training, number of instruments played, and regular music listening habits. This also served to determine whether any effect of currently playing music could be confounded by or even better explained by our other measured variables.

## 2. Materials and methods

### 2.1. Participants

To this end, we analyzed beat alignment ability scores obtained with the Computerized Adaptive Beat Alignment Test (CA-BAT; Harrison and Müllensiefen, 2018a,b) for previous studies by Spiech et al. (2022, in preparation)^1^ and Spiech et al. (2022b,c). CA-BAT scores were used either as a covariate or grouping variable while demographics information was simply used to characterize our samples (see Supplementary material 1 for an exhaustive list of all variables collected in the other three studies). In accordance with our ethics protocol approved by the Department of Psychology’s internal research ethics committee at the University of Oslo (reference number 8131575), participants provided informed consent and were compensated with gift cards of varying value. Data from 97 unique participants (46 women, 50 men, seven left handed participants) recruited for three past studies on beat synchronization to challenging “groovy” beats (Spiech et al., 2022, in preparation, see footnote 1; Spiech et al., 2022b,c) was used in this analysis. One individual did not report demographic information and we used the mlim R package (Haghish, 2022) to impute the missing observations for the machine learning regressions. Participants were 27.2 years old on average (range: 18–56, SD: 6.1 years) and listened to music for an average of 17 h per week (range: 1–84, SD: 15.1 h).

First, we classified participants into Active Musicians, Inactive Musicians, and Nonmusicians using their self-reported instruments played, musical training, and weekly music playing. Active Musicians (*N* = 48) were classified as any subjects who reported playing music weekly (M: 5.7, range: 1–27, SD: 5.9 h). Active Musicians reported receiving 10.4 years of formal musical training on average (range: 0–34, SD: 7.6 years) and played a variety of instruments (29 stringed instrumentalists, seven percussionists, four brass instrumentalists, 18 pianists, 11 vocalists, and nine other instrumentalists including electronic music producers). Inactive Musicians (*N* = 27) were classified as any subjects who reported *not* playing music weekly but had either received some musical training or reported being able to play an instrument. Inactive Musicians had an average of 5.4 years of formal musical training (range: 0–20, SD: 5.033 years) with nine playing stringed instruments, two playing percussion, five playing brass instruments, 12 playing piano, and one singing. The remaining participants (*N* = 21) reported having no musical training nor having learned to play any instrument and were thus classified as Nonmusicians (*N* = 21). These group characteristics are depicted in Table 1 below.

**Table 1:** Summary statistics of the different Musicianship groups.

Musicianship group	Number of participants	Hours played weekly	Years of formal musical training	Number of instruments played	Gender	Age
Active musicians	48	5.7 (1–27)	10.4 (0–34)	1.7 (1-4)	19 women, 29 men	27.4 (18–56)
Inactive musicians	27	0	5.4 (0–20)	1.1 (0-2)	15 women, 12 men	27.1 (20–45)
Nonmusicians	21	0	0	0	12 women, 9 men	26.5 (20–39)

For hours played weekly, years of formal musical training, and number of instruments played, the first values represent the group average while the values in parentheses are the range.

### 2.2. Procedure

For the purposes of comparing uniform data, only the information from the custom-made musicianship questionnaire (results of which are summarized in section 2.1) and from the CA-BAT were used. All three experiments began with participants filling out the demographics and musicianship questionnaire. Specifically, they provided their age, handedness, gender, years of formal musical training (specified as formal training involving a teacher or tutor), hours of weekly music playing, hours of weekly music listening, and type of instruments played (stringed, percussion, brass, piano, voice, and other which included electronic music production). Hours of weekly music listening along with type and number of instruments played were included as measures of musical engagement since more regular exposure to music and familiarity with more instruments could indicate greater musical interest. In Spiech et al. (2022, in preparation, see footnote 1) and Spiech et al. (2022b), the CA-BAT was completed at the end of each experiment whereas it was completed midway through the experiment in Spiech et al. (2022c) as part of the counterbalancing scheme. Potential effects of participant fatigue on CA-BAT performance were assessed and deemed unlikely, see Supplementary material 2 for details.

The CA-BAT is a reliable and valid psychoacoustic test that measures participants’ ability to discriminate fine differences in the timing of a musical beat (Iversen and Patel, 2008; Grahn and Schuit, 2012; Leow et al., 2014; Ross et al., 2018; Harrison and Müllensiefen, 2018a,b; Tranchant et al., 2021; Spiech et al., 2022b,c). The CA-BAT achieves this by playing 25 short musical clips with overlaid beep tracks. Each clip is played twice, once with the beep track aligned to the beat and once where the beep track is misaligned (by a constant proportion) to some extent. Participants are then asked to select the clip where they thought the beep track was aligned to the beat. The degree of misalignment is determined to some extent by participants’ accuracy where correct responses result in smaller misalignments on the subsequent trials and incorrect responses result in greater misalignments. Owing to item response theory and its adaptive design (i.e., correct responses result in smaller differences between beep tracks while incorrect responses result in greater differences), the test itself only takes around 10 min to estimate a participant’s beat alignment ability.

### 2.3. Statistical analysis

First, a Kruskal–Wallis rank sum test (a nonparametric one-way analysis of variance) with Beat Alignment Ability as the dependent variable was used to assess Musicianship (Active vs. Inactive vs. Nonmusician) group differences (Kruskal and Wallis, 1952). Follow-up two-tailed Welch’s independent samples *t*-tests were then used to test for differences in Beat Alignment Ability between groups because the variances between groups were expected to be unequal (Delacre et al., 2017). These tests were corrected for multiple comparisons using the false discovery rate (FDR, Benjamini and Hochberg, 1995). Second, to investigate the degree to which any of these differences could be related to disparities in musical training, we repeated the same tests with Years of Formal Musical Training as the dependent variable. Data analyses were carried out in R version 4.1.3 (R Core Team, 2013) using the “ez” and “effectsize” packages (Lawrence, 2011; Ben-Shachar et al., 2020) and results were visualized using the “ggplot2” package (Wickham, 2016).

Lastly, to fully explore the relationships between demographic and music-related variables with Beat Alignment Ability, we performed a generalized linear regression with all measured variables (participants’ age, gender, handedness, years of formal musical training, number of musical instruments played, number of hours of weekly music playing, and number of hours of weekly music listening) as independent variables to predict CA-BAT scores. However, we expected the music-related variables to be highly correlated with one another, potentially running into multicollinearity problems, so we additionally employed non-parametric and non-linear regression models with Gradient Boosting Machine (GBM, Friedman, 2001), Random Forest (RF, Breiman, 2001), and Extreme Gradient Boosting (XGBoost, Chen et al., 2015; Chen and Guestrin, 2016) algorithms. Tree-based algorithms such as GBM, RF, and XGBoost are *not* prone to collinearity and can effectively rank the importance of the predictors based on reduction of residual deviance or gains in other loss functions, while taking interactions between the variables into account. In this way, we sought to identify the most important factors related to beat alignment ability by extracting estimated variable importance from the model to further examine whether state-of-the-art non-parametric machine learning models would confirm the results of the generalized linear regression analysis or identify effects masked by multicollinearity.

The variable importance was estimated by the loss function gains in the process of constructing the trees. Interpreting variable importances is not necessarily analogous to correlation or regression coefficients. Instead, they can be conceptualized as variables that feed the model with unique information to improve its performance. To simplify the interpretation of variable importance, they are often scaled by dividing all estimated variable importances by the value of the variable with the highest importance. Therefore, the scaled variable importance can range from 0 to 1, ranking the importance of the predictors to the model. We used the h2o.ai software to carry out the machine learning analysis (Click et al., 2017).

## 3. Results

**Figure 1:**
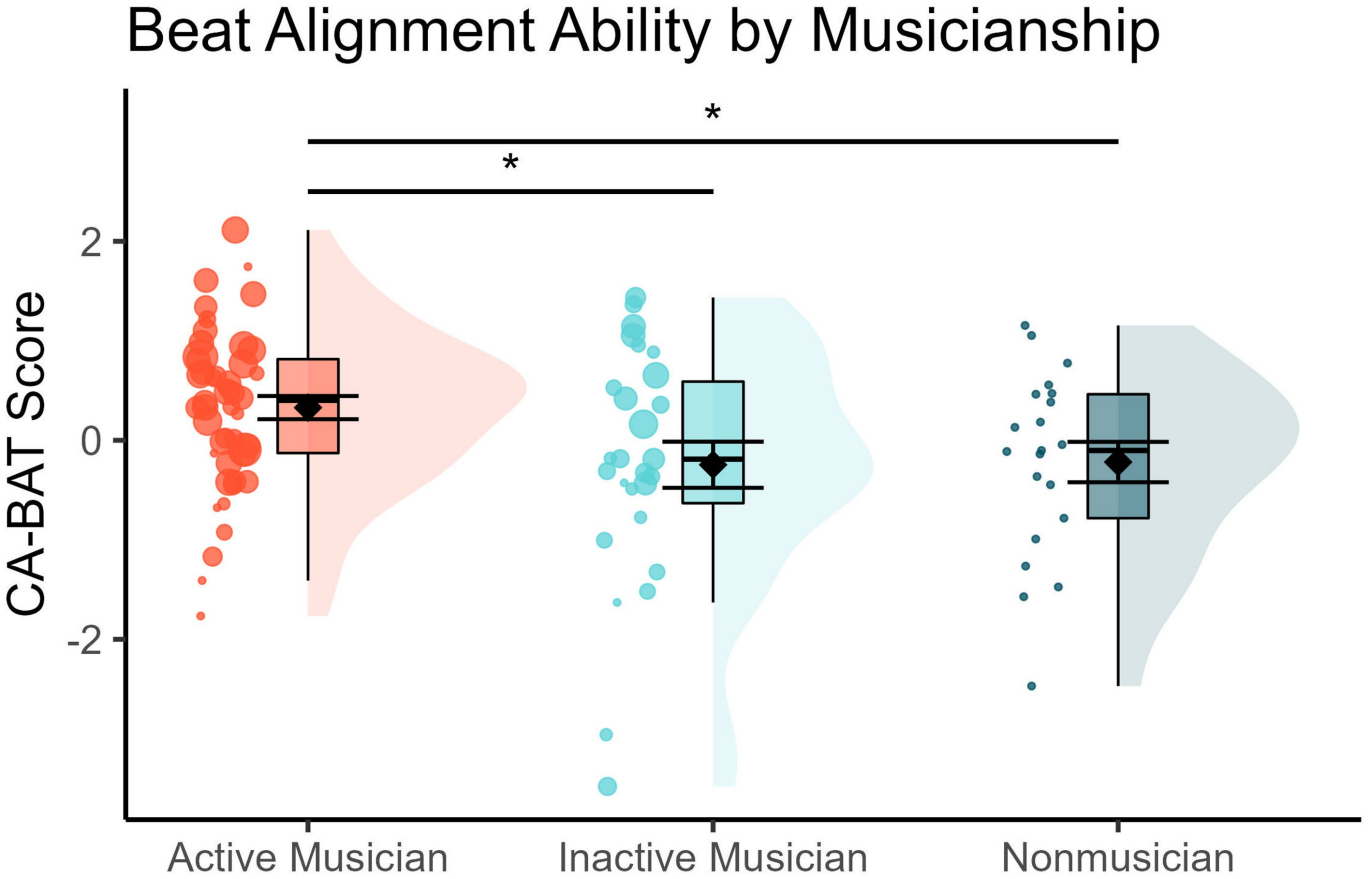
Raincloud plots displaying Beat Alignment Ability scores by Musicianship. Dots are individual subject scores and are scaled in size relative to years of formal musical training while large diamonds are group averages. Error bars represent standard errors of the mean. The boxplots’ thick black lines correspond to the group medians, hinges to the first and third quartiles, and whiskers to the most extreme value no further than 1.5 times the interquartile range. Asterisks depict statistical significance at *p* < 0.05.

The Kruskal–Wallis test with Beat Alignment Ability as a dependent variable revealed a significant effect of Musicianship [χ^2^(2) = 6.783, *p* = 0.034, ε^2^ = 0.071]. FDR-corrected follow-up two-tailed Welch’s independent samples *t*-tests revealed that Active Musicians exhibited moderately greater Beat Alignment Ability than Inactive Musicians [*t*(39.442) = 2.213, *p* = 0.050, *d* = 0.56] and even greater Beat Alignment Ability than Nonmusicians [*t*(33.82) = 2.337, *p* = 0.050, *d* = 0.63]. Inactive Musicians’ Beat Alignment Ability, on the other hand, did not differ from that of Nonmusicians [*t*(46) = -0.087, *p* = 0.931]. These results are displayed in Figure 1 below. However, when this same analysis was conducted with three outliers removed (subjects with Beat Alignment Ability scores more than ±2.5 standard deviations from the dataset’s mean), the omnibus effect was diminished to a trend [χ^2^(2) = 4.818, *p* = 0.090, ε^2^ = 0.052] and no *post hoc* pairwise comparisons survived multiple comparisons corrections (all corrected *p-*values > 0.144) so this result should be taken with caution.

Unsurprisingly, Years of Formal Musical Training also differed between Musician groups as revealed by a Kruskal–Wallis test [χ^2^(2) = 44.690, *p* < 0.001, ε^2^ = 0.470]. FDR-corrected follow-up two-tailed Welch’s independent samples *t*-tests demonstrated that all groups differed from each other with the largest differences being both musician groups having substantially more musical training than Nonmusicians [Active Musicians: *t*(47) = 9.448, *p* < 0.001, *d* = 1.93; Inactive Musicians: *t*(26) = 5.621, *p* < 0.001, *d* = 1.53], indicating that our grouping factor accounted for a difference. However, Active Musicians also had more Years of Formal Musical Training than Inactive Musicians [*t*(70.997) = 3.378, *p* = 0.001, *d* = 0.77], potentially confounding our findings about music playing and necessitating the subsequent regression analyses.

**Figure 2:**
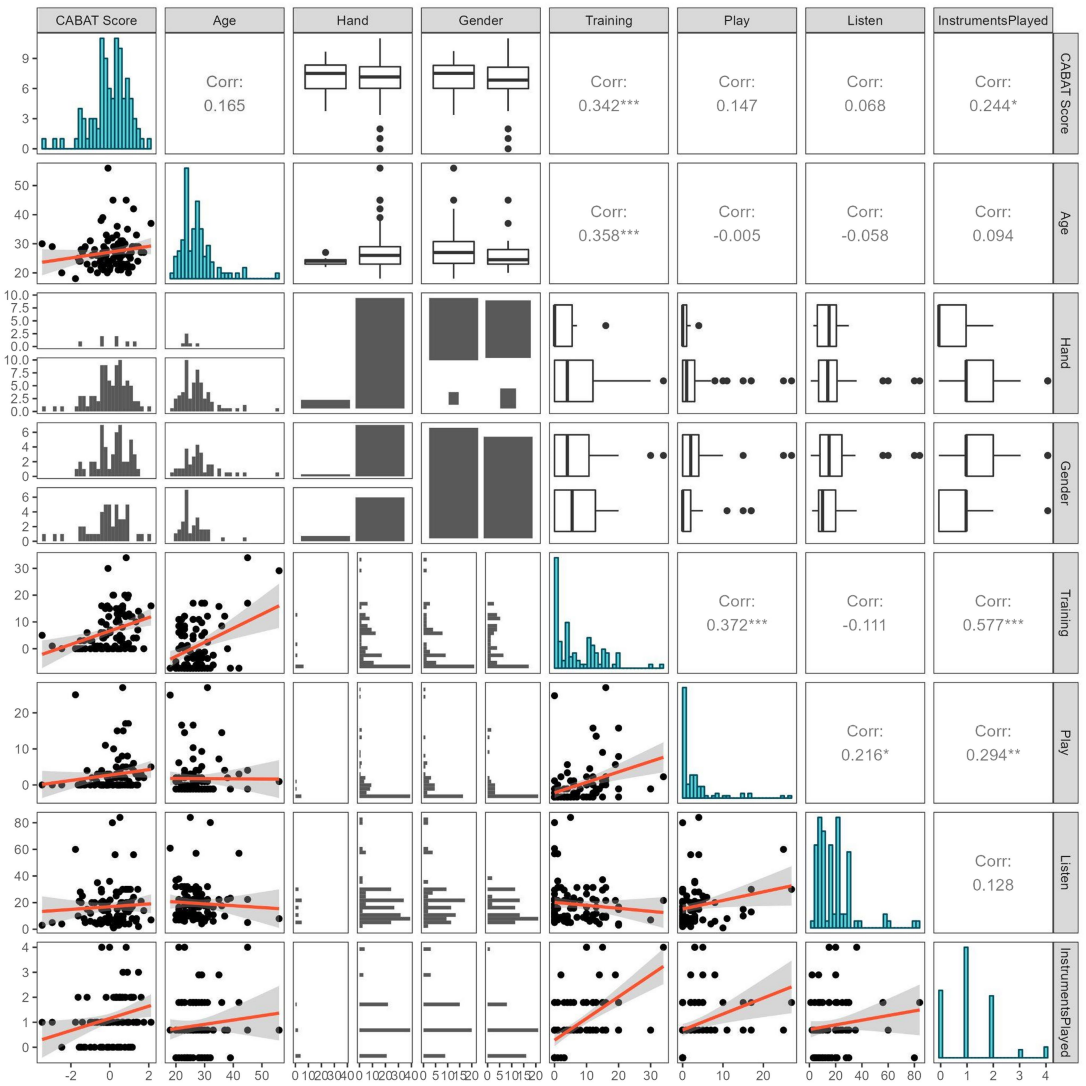
Pairs plot demonstrating the correlations between all of the different variables used in the regression analyses. As expected, and potentially troublesome for linear regression, many music-related variables were correlated with each other.

In order to rule out this potential confound, we consulted our generalized linear model’s results. The regression analysis had an R^2^ of 0.147 and mean residual deviance of 0.820 and showed that only Years of Music Training was a significant positive predictor of Beat Alignment Ability. Thus, after accounting for the variance explained by Years of Formal Musical Training, it seems that Hours of Weekly Music Playing had no bearing on Beat Alignment Ability. Table 2 presents the coefficients, standard errors, *p-*values, and standardized coefficients of the GLM predictors. The pairs plot in Figure 2 demonstrates the correlations between all of the variables. As expected, many of the music-related variables were significantly correlated which could potentially introduce multicollinearity issues for the generalized linear model, necessitating our machine learning regressions.

**Table 2:** Output of the generalized linear model. Only Years of Formal Musical Training was significant, indicating that with more years of formal musical training, CA-BAT scores increased and that once this was accounted for, no music-related or demographic variables had any impact.

Predictor	Coefficient	Standard Error	*p*-value	Standardized Coefficient
Age	0.006	0.019	0.322	0.036
Handedness	0.322	0.382	0.400	0.084
Gender	−0.207	0.219	0.348	−0.103
Years of formal musical training	0.046	0.020	0.024*	0.335
Hours of weekly music playing	−0.002	0.020	0.938	−0.009
Hours of weekly music listening	0.006	0.007	0.406	0.087
Number of instruments played	0.022	0.134	0.871	0.021

Fine-tuning the GBM, RF, and XGBoost models confirmed that the linear model was not confounded by multicollinearity problems by providing similar evidence. Extracting and rescaling the variable importance measure from the models revealed that Years of Formal Musical Training was the most important predictor of Beat Alignment Ability, explaining more of the variance compared to Number of Instruments Played, Hours of Weekly Music Listening, and particularly, Hours of Weekly Music Playing. The mean residual deviance of the fine-tuned GBM, RF, and XGBoost models were 0.65, 1.00, and 0.70, respectively. This indicates that the GBM model was the most accurate, followed by XGBoost and RF because the lower the mean residual deviance, the lower the prediction error and thus the more accurate the model. As shown in Table 3, Years of Formal Musical Training was the single most important predictor for all models, confirming the results of the generalized linear model, while handling the potential multicollinearity issues.

**Table 3:** Scaled variable importance of GBM, RF, and XGBoost models.

Variable	GBM	RF	XGBoost
Years of formal musical training	1.00	1.00	1.00
Age	0.65	0.92	0.60
Hours listened weekly	0.61	0.83	0.88
Hours played weekly	0.33	0.53	0.60
Gender	0.14	0.24	0.07
Number of instruments played	0.12	0.31	0.45
Handedness	0.01	0.08	0.09

Looking at the ranking of the variable importance indicates that Years of Formal Musical Training is the most important predictor of beat alignment for all machine learning models. Furthermore, Hours Played Weekly was relatively less important to all models, once Years of Formal Musical Training, Age, and Hours Listened Weekly are taken into consideration.

## 4. Discussion

In this study, we compared the beat alignment abilities of active musicians to those of inactive musicians and nonmusicians. We found that active musicians possessed significantly greater beat alignment abilities than both inactive musicians and nonmusicians (the latter two groups performed similarly on the CA-BAT). However, the distribution of years of formal musical training differed between groups, possibly confounding the observed beat alignment differences. The subsequent generalized linear regression indicated this had indeed occurred; years of formal musical training was the only significant predictor of CA-BAT scores. To ensure that the generalized linear model was untainted by multicollinearity problems arising from our significantly correlated music-related variables, we employed three different machine learning regressions that are not prone to these concerns in order to rank the importance of our predictors. All three machine learning regressions confirmed this was the case; years of formal musical training dwarfed all other music-related and demographic factors. These findings suggest that more musical training is associated with better beat alignment even without regular rehearsal.

This explanation falls in line with common notions of expertise where practice enhances ability (Ericsson and Lehmann, 1996; Sloboda et al., 1996). The active musicians in our sample received more years of formal musical training and likely accrued more hours of beat perception refinement through their continued engagement with their instruments, resulting in better performance on the CA-BAT than both their inactive counterparts and nonmusicians. However, these marginal effects of continued practice were superfluous for sharpening beat alignment since the regression analyses demonstrated that years of formal musical training sufficiently explained differences in CA-BAT scores. Thus, it seems that with enough training, the neural circuits for beat alignment could become hardwired and continued musical engagement is not necessary to preserve the ability.

A part of this picture could be that people with better beat alignment might be more motivated to stick with musical training for more years, further exercising their rhythmic skills. This is supported by Albert Bandura’s self-efficacy theory where one’s beliefs about one’s competencies influences subsequent motivation and performance (Bandura, 1982, 1997). This has already been shown in the context of music performance (McPherson and McCormick, 2006; Hendricks, 2016) and so it could potentially apply to lower level musical abilities as well. It seems logical that better beat alignment could result in more of Bandura’s “mastery experiences” while training, which then motivates them to pursue more formal musical education and learn more rhythmically challenging pieces in a virtuous circle. Indeed, this explanation is consistent with genetics studies that have found interactions with or no effect of practice on musical aptitude and achievement (Mosing et al., 2014; Hambrick and Tucker-Drob, 2015; Wesseldijk et al., 2019).

As to the absence of differences between active and inactive musicians, methodological aspects should also be considered. For example, it could be that the CA-BAT may not be sensitive to finding beat perception differences that arise from musical engagement factors like regular playing. Moreover, the CA-BAT measures the ability to detect fine-grained phase offsets and this is often not necessary for many instruments in a variety of musical traditions. Indeed, the perceptual center (when a sound’s onset is perceived) has been shown to vary considerably depending on a number of musical qualities (Danielsen et al., 2019), how it is measured (London et al., 2019), and genre expertise (Danielsen et al., 2022). It is possible that only highly trained musicians develop an enhanced beat perception ability that generalizes across sounds well enough to be observed in the beat alignment measured by the CA-BAT. Said another way, the CA-BAT may not be ecologically valid for untrained listeners. Other rhythmic tests should thus be employed to investigate whether this may have been the case.

Additionally, the CA-BAT’s two-alternative forced choice design introduces cognitive demands on working memory that may explain dissociations with beat tapping and production abilities (Bégel et al., 2017; Fiveash et al., 2022). These cognitive demands could be correlated with latent educational or genetic variables, that is, more years of formal musical training could be associated with more years of education in general or certain genetic predispositions (Bartholomew et al., 2015; Mosing et al., 2016). The latter could not be studied within the framework of the present study. However, with genetics alone explaining roughly 13%–16% of beat synchronization abilities (Niarchou et al., 2022), for example, this may explain why the predictors in our generalized linear model only explained about 15% of the CA-BAT scores’ variance.

A substantial limitation of this study is that our dataset did not contain potentially important details about participants’ musicianship because it was not the focus of the original studies where the data was collected. One such detail is the age that musical training began. A sensitive period for musical ability has been proposed (Penhune, 2011; Bailey and Penhune, 2013); early-trained musicians have been found to exhibit greater sensorimotor synchronization performance (Watanabe et al., 2007; Bailey and Penhune, 2010; Bailey et al., 2014) and executive functioning (Chen et al., 2022) alongside neuroanatomical differences (Amunts et al., 1997; Imfeld et al., 2009; Steele et al., 2013; Bailey et al., 2014; Shenker et al., 2022). It could be possible that only early-trained musicians (who could then accrue more years of formal musical training overall) develop an enduring beat perception while those who began their training outside of the sensitive period may either fail to cultivate better beat perception than nonmusicians or lose any gains they may have made after they stopped playing music. In our study, it is unclear to what extent our results are driven by early training so further experiments are needed to rule this out.

We also did not collect information regarding the type or quality of the formal training received. Given that musical training was the single most important predictor of CA-BAT scores we measured, it would be interesting to explore the quality of this training in future studies. For instance, it is conceivable that more intense training (i.e., more hours spent practicing) could induce more enduring beat perception abilities later in life. Furthermore, some types of musical training (e.g., private lessons, training in large or small ensembles, rigorous self-teaching) may be better or worse at enhancing beat alignment. Finally, certain musical styles and traditions require more precise beat timing than others (e.g., math rock requires better timing abilities than ambient soundscapes) so musicians trained in these genres could plausibly develop enhanced beat perception to meet their needs. Longitudinal and intervention-based studies manipulating and controlling for these various factors should thus be carried out to conclusively rule out the influence of these variables.

Finally, despite our clear instructions that formal musical training required supervision, two participants may have misunderstood what constitutes formal musical training and responded with an inordinate number of years. Removing them from the analyses, however, did not change the overall pattern of results and even when more extreme exclusion criteria were employed (see Supplementary material 3 for details).

In conclusion, from our sample of nearly a hundred participants, we found that better beat alignment ability was associated with more years of formal musical training and that this relationship dwarfed any potential effect of currently playing music. This was confirmed using both a conventional generalized linear model as well as nonlinear and nonparametric machine learning regressions that are not prone to the multicollinearity issues that may arise while measuring typically intercorrelated musical variables. However, since our model only captured roughly 15% of the variance in our data, future work is needed to tease out the exact nature and causal relations of these variables. This could include using other rhythmic tests, genetic information, longitudinal designs, and more detailed demographics questionnaires (e.g., type of musical training and age at which musical training began). Further, to definitively rule out potential pre-selection effects found in previous work (i.e., innately talented musicians continue training longer than those without such talent), intervention studies are needed to test whether training can improve the rhythmic abilities of participants of varying natural skill.

## Data availability statement

Publicly available datasets were analyzed in this study. This data can be found at: https://osf.io/d6mje/?view_only=d0910426b5eb481d9cff1850c4f874f6.

## Ethics statement

The studies involving human participants were reviewed and approved by Internal Ethics Committee, Department of Psychology University of Oslo reference number: 8131575. The patients/participants provided their written informed consent to participate in this study.

## Author contributions

CS conceived the study and carried out the statistical analyses and data visualizations with EH. CS wrote the first draft of the manuscript. TE, BL, AD, and EH all provided feedback and revised the manuscript. All authors contributed to the article and approved the submitted version.

## Funding

This work was partially supported by the Research Council of Norway through its Centres of Excellence scheme, project number 262762.

## Conflict of interest

The authors declare that the research was conducted in the absence of any commercial or financial relationships that could be construed as a potential conflict of interest.

## Publisher’s note

All claims expressed in this article are solely those of the authors and do not necessarily represent those of their affiliated organizations, or those of the publisher, the editors and the reviewers. Any product that may be evaluated in this article, or claim that may be made by its manufacturer, is not guaranteed or endorsed by the publisher.

## Footnotes

^1^Spiech, C., Sioros, G., Danielsen, A., Laeng, B., and Endestad, T. (2022). Oscillatory Attention in Groove (in preparation).

## References

[ref1] Amunts K. Schlaug G. Jäncke L. Steinmetz H. Schleicher A. Dabringhaus A.. (1997). Motor cortex and hand motor skills: structural compliance in the human brain. Hum. Brain Mapp. 5, 206–215. doi: 10.1002/(SICI)1097-0193(1997)5:3<206::AID-HBM5>3.0.CO;2-7, PMID: 20408216

[ref2] Appelgren A. Osika W. Theorell T. Madison G. Bojner Horwitz E. (2019). Tuning in on motivation: differences between non-musicians, amateurs, and professional musicians. Psychol. Music 47, 864–873. doi: 10.1177/0305735619861435

[ref3] Bailey J. A. Penhune V. B. (2010). Rhythm synchronization performance and auditory working memory in early- and late-trained musicians. Exp. Brain Res. 204, 91–101. doi: 10.1007/s00221-010-2299-y, PMID: 20508918

[ref4] Bailey J. A. Penhune V. B. (2013). The relationship between the age of onset of musical training and rhythm synchronization performance: validation of sensitive period effects. Front. Neurosci. 7:227. doi: 10.3389/fnins.2013.00227, PMID: 24348323 PMC3843222

[ref5] Bailey J. A. Zatorre R. J. Penhune V. B. (2014). Early musical training is linked to gray matter structure in the ventral premotor cortex and auditory–motor rhythm synchronization performance. J. Cogn. Neurosci. 26, 755–767. doi: 10.1162/jocn_a_00527 24236696

[ref6] Bandura A. (1982). Self-efficacy mechanism in human agency. Am. Psychol. 37, 122–147. doi: 10.1037/0003-066X.37.2.122

[ref7] Bandura A. (1997). Self-efficacy: The Exercise of Control. New York, US: W.H. Freeman.

[ref8] Bartholomew A. J. Meck W. H. Cirulli E. T. (2015). Analysis of genetic and non-genetic factors influencing timing and time perception. PLoS One 10:e0143873. doi: 10.1371/journal.pone.0143873, PMID: 26641268 PMC4671567

[ref9] Bégel V. Benoit C.-E. Correa A. Cutanda D. Kotz S. A. Dalla Bella S. (2017). “Lost in time” but still moving to the beat. Neuropsychologia 94, 129–138. doi: 10.1016/j.neuropsychologia.2016.11.022, PMID: 27914979

[ref10] Benjamini Y. Hochberg Y. (1995). Controlling the false discovery rate: a practical and powerful approach to multiple testing. J. R. Stat. Soc. Series B 57, 289–300. doi: 10.2307/2346101

[ref11] Ben-Shachar M. Lüdecke D. Makowski D. (2020). Effectsize: estimation of effect size indices and standardized parameters. J. Open Source Softw. 5:2815. doi: 10.21105/joss.02815

[ref12] Bonde L. O. Juel K. Ekholm O. (2018). Associations between music and health-related outcomes in adult non-musicians, amateur musicians and professional musicians—results from a nationwide Danish study. Nord. J. Music Ther. 27, 262–282. doi: 10.1080/08098131.2018.1439086

[ref13] Brattico E. Näätänen R. Tervaniemi M. (2001). Context effects on pitch perception in musicians and nonmusicians: evidence from event-related-potential recordings. Music. Percept. 19, 199–222. doi: 10.1525/mp.2001.19.2.199

[ref14] Breiman L. (2001). Random forests. Mach. Learn. 45, 5–32. doi: 10.1023/A:1010933404324

[ref15] Calvo-Merino B. Ehrenberg S. Leung D. Haggard P. (2010). Experts see it all: Configural effects in action observation. Psychol. Res. 74, 400–406. doi: 10.1007/s00426-009-0262-y 19856185

[ref16] Calvo-Merino B. Glaser D. E. Grèzes J. Passingham R. E. Haggard P. (2005). Action observation and acquired motor skills: an fMRI study with expert dancers. Cereb. Cortex 15, 1243–1249. doi: 10.1093/cercor/bhi007, PMID: 15616133

[ref17] Calvo-Merino B. Grèzes J. Glaser D. E. Passingham R. E. Haggard P. (2006). Seeing or doing? Influence of visual and motor familiarity in action observation. Curr. Biol. 16, 1905–1910. doi: 10.1016/j.cub.2006.07.065 17027486

[ref18] Chen T. Guestrin C. (2016). Xgboost: a scalable tree boosting system. In: *Proceedings of the 22nd Acm Sigkdd International Conference on Knowledge Discovery and Data Mining*, 785–794.

[ref19] Chen T. He T. Benesty M. Khotilovich V. Tang Y. Cho H.. (2015). Xgboost: Extreme gradient boosting. R Package Version 0.4-2, 1–4.

[ref20] Chen J. L. Penhune V. B. Zatorre R. J. (2008). Moving on time: brain network for auditory-motor synchronization is modulated by rhythm complexity and musical training. J. Cogn. Neurosci. 20, 226–239. doi: 10.1162/jocn.2008.20018, PMID: 18275331

[ref21] Chen J. Scheller M. Wu C. Hu B. Peng R. Liu C.. (2022). The relationship between early musical training and executive functions: validation of effects of the sensitive period. Psychol. Music 50, 86–99. doi: 10.1177/0305735620978690

[ref22] Chi M. T. H. Glaser R. Farr M. J. (2014). The Nature of Expertise. New York, US: Psychology Press.

[ref23] Click C. Malohlava M. Candel A. Roark H. Parmar V. (2017). Gradient Boosting Machine with h2o. H2O. Ai. CA: H_2_O.ai, Inc.

[ref24] Cohen M. A. Evans K. K. Horowitz T. S. Wolfe J. M. (2011). Auditory and visual memory in musicians and nonmusicians. Psychon. Bull. Rev. 18, 586–591. doi: 10.3758/s13423-011-0074-0, PMID: 21374094 PMC3967744

[ref25] Criscuolo A. Pando-Naude V. Bonetti L. Vuust P. Brattico E. (2022). An ALE meta-analytic review of musical expertise. Sci. Rep. 12:11726. doi: 10.1038/s41598-022-14959-4, PMID: 35821035 PMC9276732

[ref26] Danielsen A. Nymoen K. Anderson E. Câmara G. S. Langerød M. T. Thompson M. R.. (2019). Where is the beat in that note? Effects of attack, duration, and frequency on the perceived timing of musical and quasi-musical sounds. J. Exp. Psychol. Hum. Percept. Perform. 45, 402–418. doi: 10.1037/xhp0000611 30802130

[ref27] Danielsen A. Nymoen K. Langerød M. T. Jacobsen E. Johansson M. London J. (2022). Sounds familiar (?): expertise with specific musical genres modulates timing perception and micro-level synchronization to auditory stimuli. Atten. Percept. Psychophys. 84, 599–615. doi: 10.3758/s13414-021-02393-z, PMID: 34862587 PMC8888399

[ref28] Dawson W. J. (2014). Benefits of music training are widespread and lifelong: a bibliographic review of their non-musical effects. Med. Probl. Perform. Art. 29, 57–63. doi: 10.21091/mppa.2014.2014, PMID: 24925171

[ref29] Delacre M. Lakens D. Leys C. (2017). Why psychologists should by default use Welch’s t-test instead of Student’s t-test. Int. Rev. Soc. Psychol. 30:92. doi: 10.5334/irsp.82

[ref30] Ericsson K. A. Charness N. (1994). Expert performance: its structure and acquisition. Am. Psychol. 49, 725–747. doi: 10.1037/0003-066X.49.8.725

[ref31] Ericsson K. A. Lehmann A. C. (1996). Expert and exceptional performance: evidence of maximal adaptation to task constraints. Annu. Rev. Psychol. 47, 273–305. doi: 10.1146/annurev.psych.47.1.273, PMID: 15012483

[ref32] Feltovich P. J. Prietula M. J. Ericsson K. A. (2006). Studies of Expertise from Psychological Perspectives. New York, US: Cambridge University Press.

[ref33] Fiveash A. Bella S. D. Bigand E. Gordon R. L. Tillmann B. (2022). You got rhythm, or more: the multidimensionality of rhythmic abilities. Atten. Percept. Psychophys. 84, 1370–1392. doi: 10.3758/s13414-022-02487-2, PMID: 35437703 PMC9614186

[ref34] Franěk M. Mates J. Radil T. Beck K. Pöppel E. (1991). Finger tapping in musicians and nonmusicians. Int. J. Psychophysiol. 11, 277–279. doi: 10.1016/0167-8760(91)90022-P 1797762

[ref35] Friedman J. H. (2001). Greedy function approximation: a gradient boosting machine. Ann. Stat. 29, 1189–1232. doi: 10.1214/aos/1013203451

[ref36] Gaser C. Schlaug G. (2003). Gray matter differences between musicians and nonmusicians. Ann. N. Y. Acad. Sci. 999, 514–517. doi: 10.1196/annals.1284.062, PMID: 14681175

[ref37] Grahn J. A. Brett M. (2007). Rhythm and beat perception in motor areas of the brain. J. Cogn. Neurosci. 19, 893–906. doi: 10.1162/jocn.2007.19.5.893 17488212

[ref38] Grahn J. A. McAuley J. D. (2009). Neural bases of individual differences in beat perception. Neuroimage 47, 1894–1903. doi: 10.1016/j.neuroimage.2009.04.039, PMID: 19376241

[ref39] Grahn J. A. Rowe J. B. (2009). Feeling the beat: premotor and striatal interactions in musicians and nonmusicians during beat perception. J. Neurosci. 29, 7540–7548. doi: 10.1523/JNEUROSCI.2018-08.2009, PMID: 19515922 PMC2702750

[ref40] Grahn J. A. Schuit D. (2012). Individual differences in rhythmic ability: behavioral and neuroimaging investigations. Psychomusicology 22, 105–121. doi: 10.1037/a0031188

[ref41] Haghish E. F. (2022). mlim: Multiple Imputation with Automated Machine Learning. Available at: https://CRAN.R-project.org/package=mlim

[ref42] Hambrick D. Z. Tucker-Drob E. M. (2015). The genetics of music accomplishment: evidence for gene–environment correlation and interaction. Psychon. Bull. Rev. 22, 112–120. doi: 10.3758/s13423-014-0671-9, PMID: 24957535

[ref43] Hanna-Pladdy B. Gajewski B. (2012). Recent and past musical activity predicts cognitive aging variability: direct comparison with general lifestyle activities. Front. Hum. Neurosci. 6:198. doi: 10.3389/fnhum.2012.00198, PMID: 22833722 PMC3400047

[ref44] Harrison P. M. C. Müllensiefen D. (2018a). Development and validation of the computerised adaptive beat alignment test CA-BAT. Sci. Rep. 8, 1–19. doi: 10.1038/s41598-018-30318-8, PMID: 30120265 PMC6097996

[ref45] Harrison P. M. C. Müllensiefen D. (2018b). doi: 10.5281/zenodo.1415353

[ref46] Hendricks K. S. (2016). The sources of self-efficacy: educational research and implications for music. Update Applicat. Res. Music Educ. 35, 32–38. doi: 10.1177/8755123315576535

[ref47] Hinojosa-Aguayo I. Garcia-Burgos D. Catena A. González F. (2022). Implicit and explicit measures of the sensory and hedonic analysis of beer: the role of tasting expertise. Food Res. Int. 152:110873. doi: 10.1016/j.foodres.2021.110873, PMID: 35181065

[ref48] Hove M. J. Spivey M. J. Krumhansl C. L. (2010). Compatibility of motion facilitates visuomotor synchronization. J. Exp. Psychol. Hum. Percept. Perform. 36, 1525–1534. doi: 10.1037/a0019059 20695698

[ref49] Imfeld A. Oechslin M. S. Meyer M. Loenneker T. Jancke L. (2009). White matter plasticity in the corticospinal tract of musicians: a diffusion tensor imaging study. Neuroimage 46, 600–607. doi: 10.1016/j.neuroimage.2009.02.025, PMID: 19264144

[ref50] Iversen J. R. Patel A. D. (2008). The Beat Alignment Test (BAT): Surveying Beat Processing Abilities in the General Population.

[ref51] Jäncke L. (2009). The plastic human brain. Restor. Neurol. Neurosci. 27, 521–538. doi: 10.3233/RNN-2009-0519 19847074

[ref52] Kauffman W. H. Carlsen J. C. (1989). Memory for intact music works: the importance of music expertise and retention interval. Psychomusicology 8, 3–20. doi: 10.1037/h0094235

[ref53] Kishon-Rabin L. Amir O. Vexler Y. Zaltz Y. (2001). Pitch discrimination: are professional musicians better than non-musicians? J. Basic Clin. Physiol. Pharmacol. 12, 125–144. doi: 10.1515/JBCPP.2001.12.2.125 11605682

[ref54] Krause V. Pollok B. Schnitzler A. (2010). Perception in action: the impact of sensory information on sensorimotor synchronization in musicians and non-musicians. Acta Psychol. 133, 28–37. doi: 10.1016/j.actpsy.2009.08.003, PMID: 19751937

[ref55] Kruskal W. H. Wallis W. A. (1952). Use of ranks in one-criterion variance analysis. J. Am. Stat. Assoc. 47, 583–621. doi: 10.1080/01621459.1952.10483441

[ref56] Lawrence M. A. (2011). Package ez: Easy analysis and visualization of factorial experiments. Available at: http://CRAN.R-project.org/package=ez

[ref57] Lehmann A. C. Gruber H. Kopiez R. (2018). “Expertise in music,” in The Cambridge Handbook of Expertise and Expert Performance. 2nd *Edn*. eds. Ericsson K. A. Hoffman R. R. Kozbelt A. Williams A. M. (New York, US: Cambridge University Press), 535–549.

[ref58] Lehmann A. C. Sloboda J. A. Woody R. H. (2007). Psychology for Musicians. Oxford, England, UK: Oxford University Press.

[ref59] Leow L. A. Parrott T. Grahn J. A. (2014). Individual differences in beat perception affect gait responses to low-and high-groove music. Front. Hum. Neurosci. 8:811. doi: 10.3389/fnhum.2014.00811 25374521 PMC4205839

[ref60] London J. Nymoen K. Langerød M. T. Thompson M. R. Code D. L. Danielsen A. (2019). A comparison of methods for investigating the perceptual center of musical sounds. Atten. Percept. Psychophys. 81, 2088–2101. doi: 10.3758/s13414-019-01747-y, PMID: 31077060

[ref61] Madsen C. K. (1979). Modulated beat discrimination among musicians and nonmusicians. J. Res. Music. Educ. 27, 57–67. doi: 10.2307/3344892

[ref62] McPherson G. E. McCormick J. (2006). Self-efficacy and music performance. Psychol. Music 34, 322–336. doi: 10.1177/0305735606064841

[ref63] Micheyl C. Delhommeau K. Perrot X. Oxenham A. J. (2006). Influence of musical and psychoacoustical training on pitch discrimination. Hear. Res. 219, 36–47. doi: 10.1016/j.heares.2006.05.004, PMID: 16839723

[ref64] Mikutta C. A. Maissen G. Altorfer A. Strik W. Koenig T. (2014). Professional musicians listen differently to music. Neuroscience 268, 102–111. doi: 10.1016/j.neuroscience.2014.03.007 24637097

[ref65] Mosing M. A. Madison G. Pedersen N. L. Kuja-Halkola R. Ullén F. (2014). Practice does not make perfect: no causal effect of music practice on music ability. Psychol. Sci. 25, 1795–1803. doi: 10.1177/0956797614541990 25079217

[ref66] Mosing M. A. Verweij K. J. H. Madison G. Ullén F. (2016). The genetic architecture of correlations between perceptual timing, motor timing, and intelligence. Intelligence 57, 33–40. doi: 10.1016/j.intell.2016.04.002

[ref67] Nguyen T. Gibbings A. Grahn J. (2018). “Rhythm and beat perception,” in Springer Handbook of Systematic Musicology. ed. Bader R. (Heidelberg, Germany: Springer Berlin Heidelberg), 507–521.

[ref68] Niarchou M. Gustavson D. E. Sathirapongsasuti J. F. Anglada-Tort M. Eising E. Bell E.. (2022). Genome-wide association study of musical beat synchronization demonstrates high polygenicity. Nat. Hum. Behav. 6, 1–18. doi: 10.1038/s41562-022-01359-x 35710621 PMC9489530

[ref69] Orgs G. Calvo-Merino B. Cross E. S. (2018). “Knowing dance or knowing how to dance?: sources of expertise in aesthetic appreciation of human movement,” in The Neurocognition of Dance. eds. B. Bläsing, M. Puttke and T. Schack (London, England: Routledge), 238–257.

[ref70] Orgs G. Dombrowski J.-H. Heil M. Jansen-Osmann P. (2008). Expertise in dance modulates alpha/beta event-related desynchronization during action observation. Eur. J. Neurosci. 27, 3380–3384. doi: 10.1111/j.1460-9568.2008.06271.x, PMID: 18598273

[ref71] Pallesen K. J. Brattico E. Bailey C. J. Korvenoja A. Koivisto J. Gjedde A.. (2010). Cognitive control in auditory working memory is enhanced in musicians. PLoS One 5:e11120. doi: 10.1371/journal.pone.0011120, PMID: 20559545 PMC2886055

[ref72] Penhune V. B. (2011). Sensitive periods in human development: evidence from musical training. Cortex 47, 1126–1137. doi: 10.1016/j.cortex.2011.05.010, PMID: 21665201

[ref73] R Core Team. (2013). R: A language and environment for statistical computing. In R Foundation for Statistical Computing. Available at: http://www.R-project.org/

[ref74] Rammsayer T. Altenmüller E. (2006). Temporal information processing in musicians and nonmusicians. Music. Percept. 24, 37–48. doi: 10.1525/mp.2006.24.1.37

[ref75] Repp B. H. (2010). Sensorimotor synchronization and perception of timing: effects of music training and task experience. Hum. Mov. Sci. 29, 200–213. doi: 10.1016/j.humov.2009.08.002, PMID: 20074825

[ref76] Repp B. H. Doggett R. (2007). Tapping to a very slow beat: a comparison of musicians and nonmusicians. Music. Percept. 24, 367–376. doi: 10.1525/mp.2007.24.4.367

[ref77] Romeiser J. L. Smith D. M. Clouston S. A. P. (2021). Musical instrument engagement across the life course and episodic memory in late life: an analysis of 60 years of longitudinal data from the Wisconsin longitudinal study. PLoS One 16:e0253053. doi: 10.1371/journal.pone.0253053, PMID: 34166389 PMC8224921

[ref78] Ross J. M. Iversen J. R. Balasubramaniam R. (2018). The role of posterior parietal cortex in beat-based timing perception: a continuous theta burst stimulation study. J. Cogn. Neurosci. 30, 634–643. doi: 10.1162/jocn_a_01237, PMID: 29346017

[ref79] Schulze H.-H. (1978). The detectability of local and global displacements in regular rhythmic patterns. Psychol. Res. 40, 173–181. doi: 10.1007/BF00308412, PMID: 693733

[ref80] Shea J. B. Paull G. (2014). “Capturing expertise in sports,” in The Road to Excellence (New York, US: Psychology Press), 321–335.

[ref81] Shenker J. J. Steele C. J. Chakravarty M. M. Zatorre R. J. Penhune V. B. (2022). Early musical training shapes cortico-cerebellar structural covariation. Brain Struct. Funct. 227, 407–419. doi: 10.1007/s00429-021-02409-2, PMID: 34657166

[ref82] Shors T. J. Anderson M. L. Curlik Ii D. Nokia M. (2012). Use it or lose it: how neurogenesis keeps the brain fit for learning. Behav. Brain Res. 227, 450–458. doi: 10.1016/j.bbr.2011.04.023, PMID: 21536076 PMC3191246

[ref83] Skaansar J. F. Laeng B. Danielsen A. (2019). Microtiming and mental effort: onset asynchronies in musical rhythm modulate pupil size. Music. Percept. 37, 111–133. doi: 10.1525/mp.2019.37.2.111

[ref84] Sloboda J. A. (1991). “Musical expertise,” in Toward a General Theory of Expertise: Prospects and Limits. eds. K. Anders Ericsson and J. Smith (New York, US: Cambridge University Press), 153–171.

[ref85] Sloboda J. A. Davidson J. W. Howe M. J. A. Moore D. G. (1996). The role of practice in the development of performing musician. Br. J. Psychol. 87, 287–309. doi: 10.1111/j.2044-8295.1996.tb02591.x

[ref86] Spiech C. Hope M. Câmara S. Guilherme S. George E. Tor M.. (2022c). Sensorimotor synchronization increases groove. [Preprint]. doi: 10.5281/ZENODO.6908098

[ref88] Spiech C. Sioros G. Endestad T. Danielsen A. Laeng B. (2022b). Pupil drift rate indexes groove ratings. Sci. Rep. 12:11620. doi: 10.1038/s41598-022-15763-w, PMID: 35804069 PMC9270355

[ref89] Steele C. J. Bailey J. A. Zatorre R. J. Penhune V. B. (2013). Early musical training and white-matter plasticity in the corpus callosum: evidence for a sensitive period. J. Neurosci. 33, 1282–1290. doi: 10.1523/JNEUROSCI.3578-12.2013, PMID: 23325263 PMC6704889

[ref90] Sternberg R. J. Grigorenko E. L. (2003). The Psychology of Abilities, Competencies, and Expertise. New York, US: Cambridge University Press.

[ref91] Talamini F. Altoè G. Carretti B. Grassi M. (2018). Correction: musicians have better memory than nonmusicians: a meta-analysis. PLoS One 13:e0191776. doi: 10.1371/journal.pone.0191776, PMID: 29352315 PMC5774829

[ref92] Tempere S. de Revel G. Sicard G. (2019). Impact of learning and training on wine expertise: a review. Curr. Opin. Food Sci. 27, 98–103. doi: 10.1016/j.cofs.2019.07.001

[ref93] Tervaniemi M. Just V. Koelsch S. Widmann A. Schrger E. (2005). Pitch discrimination accuracy in musicians vs nonmusicians: an event-related potential and behavioral study. Exp. Brain Res. 161, 1–10. doi: 10.1007/s00221-004-2044-5, PMID: 15551089

[ref94] Tranchant P. Lagrois M.-É. Bellemare A. Schultz B. G. Peretz I. (2021). Co-occurrence of deficits in beat perception and synchronization supports implication of motor system in beat perception. Music Sci. 4:205920432199171. doi: 10.1177/2059204321991713

[ref95] Watanabe D. Savion-Lemieux T. Penhune V. B. (2007). The effect of early musical training on adult motor performance: evidence for a sensitive period in motor learning. Exp. Brain Res. 176, 332–340. doi: 10.1007/s00221-006-0619-z, PMID: 16896980

[ref96] Wesseldijk L. W. Mosing M. A. Ullén F. (2019). Gene–environment interaction in expertise: the importance of childhood environment for musical achievement. Dev. Psychol. 55, 1473–1479. doi: 10.1037/dev0000726, PMID: 30883154

[ref97] Wickham H. (2016). ggplot2: Elegant Graphics for Data Analysis. 2nd *Edn*. New York, US: Springer International Publishing, Imprint, Springer.

