# Supplementary material for: Beat alignment ability is associated with formal musical training not current music playing
Source: Front Psychol. 2023 Jan 30;14:1034561. doi: 10.3389/fpsyg.2023.1034561 (PMC9922839; doi:10.3389/fpsyg.2023.1034561)
Supplement: Supplementary file 1 [file Data_Sheet_1.pdf]

Supplementary Material 1. Variables Collected in the Source Studies

As noted in the main text, demographics data were collected in three previous studies to characterize our samples and CA-BAT scores were used as a covariate or grouping variable. In the first study, participants were split into high and low CA-BAT performance using a median split and this was then used as a grouping variable for our analyses testing the effects of rhythmic complexity on both groove ratings and pupil drift rate (Spiech et al., 2022). In the second study, a correlation matrix of all measured variables (demographics information, CA-BAT scores, tapping variability, tapping intensity, groove ratings, and pupil dilation) was used to determine covariates. After correcting for multiple comparisons, a significant correlation between tapping variability and CA-BAT score was observed and so CA-BAT scores were used as a between-subjects factor alongside the within-subject factor rhythmic complexity in a mixed analysis of variance with tapping variability as the dependent variable (Spiech, Connor, Hope, Mikael, et al., 2022). In the final experiment, CA-BAT scores will be used in a similar manner as in the second study; a correlation matrix will be used to determine which dependent variables should be investigated using CA-BAT scores as a covariate. Measured variables in this study include: evoked pupil dilation, pupil phase coherence, EEG phase coherence, and groove ratings (Spiech, Connor, Sioros, George, et al., 2022).

Supplementary Material 2. Potential Effects of Participant Fatigue

Given that the CA-BAT was administered at different points in the three different experiments, it seems possible that differing levels of fatigue could have impacted our results. In the first experiment, the CA-BAT was administered at the end of the study, around 40 minutes; in the second experiment, the CA-BAT was administered halfway through the study around 30

minutes in; and in the third experiment, the CA-BAT was administered at the end of the experiment, around 80-90 minutes. However, upon further investigation, this seems unlikely. Plotting the results by Experiment (1, 2, and 3) seem to depict differences unlikely to result from fatigue (see Supplementary Figure 1 below). For instance, Nonmusicians in Experiment 3 seemed to score slightly better than in Experiment 1 despite taking almost twice as long to complete a more boring experiment (three of the six drumbeats from Experiment 1 were repeated for an hour in Experiment 3). Further, in Experiment 2 where subjects would have been the least fatigued, Inactive Musicians seem to have performed somewhat worse than in Experiments 1 and 3 despite Active Musicians performing similarly across all three Experiments. Instead, these messier results are more likely a result of random variance associated with such small sample sizes, especially in the Nonmusician groups from Experiments 1 and 3 with only 5 and 6 subjects, respectively. Therefore, we pooled all of the data together and employed nonparametric statistics to account for this.

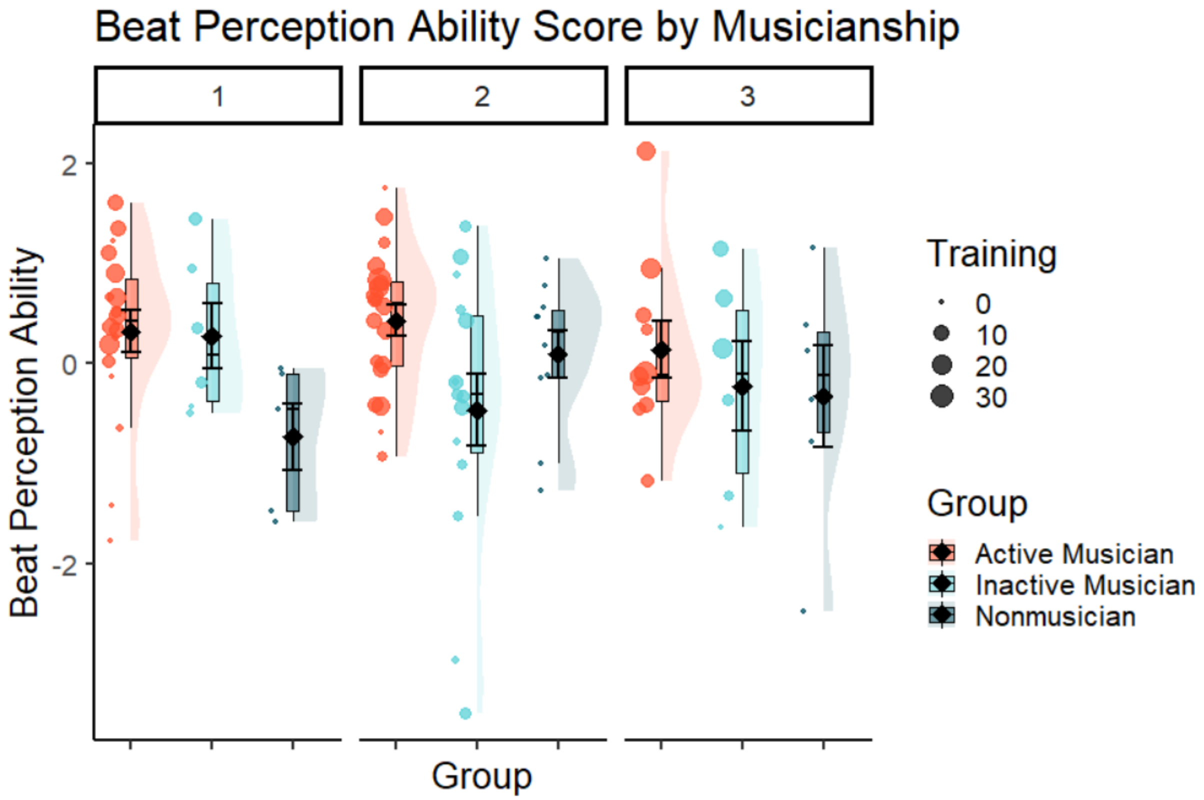

Supplementary Figure 1. Musicianship group analyses split by experiment.

However, plots can be deceiving and so to be absolutely certain that our results weren't confounded by fatigue effects, we re-ran the Kruskal-Wallis tests on each of the three datasets separately. All tests were insignificant. If there was an effect of fatigue in one of the experiments but not the others, then we should have seen different results for each experiment (e.g., a significant effect in Experiment 2 where people were least fatigued and no effect in Experiment 3 where people were most fatigued). This could be due to the poorer statistical power and more drastically unbalanced nature of the groups or it could validate the null results we observed when outliers were removed. Finally, as a last check, we also re-ran the generalized linear model with Experiment included as a predictor to ensure that our exploratory analyses weren't confounded either. The results only changed slightly; the original R^2 of 0.147 increased to 0.159 and Years of

Musical Training remained the only significant predictor with a slightly lower *p*-value of 0.020 (compared to the original 0.024) and a slightly higher estimate of 0.049 (compared to the original 0.046).

Supplementary Material 3. Control Analysis with Musical Training Outliers Excluded

Several of the participants reported years of musical training that some may find implausible (e.g., a subject reported 30 years of musical training and another reported 34 years). Thus, it could be possible that these subjects misunderstood the question. To demonstrate that this only minimally impacted our results, we re-ran both the Kruskal-Wallis tests and the generalized linear model without the participants who reported years of musical training greater than two and a half standard deviations more than the median. The Kruskal-Wallis tests were largely unchanged, albeit slightly weaker ($\chi^2(2)=6.783$, $p=0.034$, $\varepsilon^2=0.071$ with the two musical training outliers and $\chi^2(2)=6.474$, $p=0.039$, $\varepsilon^2=0.069$ without them). Similarly, the results of the GLM did not fundamentally change. In fact, the effect of Years of Musical Training increased somewhat (from $b=0.046$, 95% CI [0.006, 0.086], $p=0.024$ with the two musical training outliers to $b=0.068$, 95% CI [0.025, 0.111], $p=0.003$ without them). Furthermore, the model fit increased (from an R^2 of 0.147 to 0.192).

A secondary concern may be that participants with many years of formal musical training may have been filtered by entrance exams into post-secondary music schools. To ensure that this only minimally impacted our results, we took an extremely conservative approach and excluded all subjects with more than 12 years of formal musical training (i.e., the maximum years of formal musical training our participants could have received purely within their primary

education). The effect of formal musical training in the GLM became even more pronounced (b=0.103, 95% CI [0.033, 0.172], p=0.005), indicating that this potential confound was unlikely.

Further, to demonstrate that the effect was not driven by pooling participants drawn from different populations (musicians with varying predictor values and nonmusicians with zero variance), we repeated the GLM without the Nonmusicians. The GLM still reveals only an effect of Years of Formal Musical Training (b=0.053, 95% CI [.010, 0.096], p=0.017) as shown in Supplementary Figure 2 below.

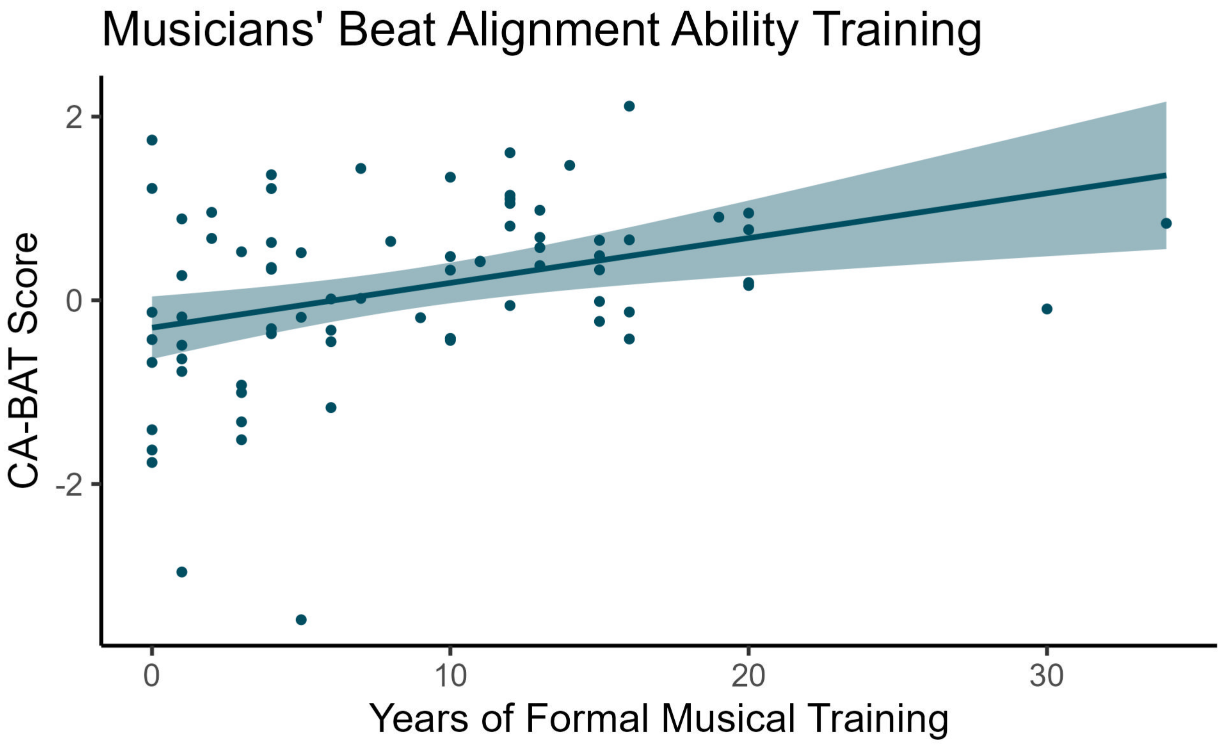

Supplementary Figure 2. Significant effect of Years of Formal Musical Training with Nonmusicians excluded.

As an additional precaution and rough proxy for primary vs. post-secondary musical education comparisons, we took the remaining 75 participants and split them into High (N=21)

and Low Training (N=54) groups based on the 12 year cutoff. When this new grouping factor was added to the GLM and allowed to interact with Years of Formal Musical Training, we found that the Low Training group performed worse on the CA-BAT (b=-2.147, 95% CI [-3.872, -0.422], p=0.017; Kruskal-Wallis test: $\chi^2(2)$=4.869, p=0.027, ε^2=0.065) and that there was a significant interaction between Years of Formal Musical Training and Training Level (b=0.164, 95% CI [0.046, 0.281], p=0.008). This interaction was driven by the Low Training group who exhibited an effect of Years of Formal Musical Training (b=0.117, 95% CI [0.042, 0.192], p=0.004) in opposition to the High Training group who did not (b=-0.034, 95% CI [-0.121, 0.053], p>0.4).

Taking both of these effects into account, this could indicate that either formal musical training confers no additional benefits to CA-BAT performance after around 12 years or that the highly skilled participants were screened for or predisposed to enroll in additional training. However, one probably shouldn't put much stock into these results since the High Training group did not, on average, perform exceptionally high on the CA-BAT (only 0.536), perhaps because its sample was less than half that of the Low Training group. It's quite possible that these results would change with more data as well as basing the grouping factor directly on participants' reports describing their formal musical training (i.e., post-secondary vs. exclusively primary school music education). The distribution of participants across groups, their CA-BAT scores, and their individual regression lines are plotted below in Supplementary Figures 3, 4, and 5, respectively.

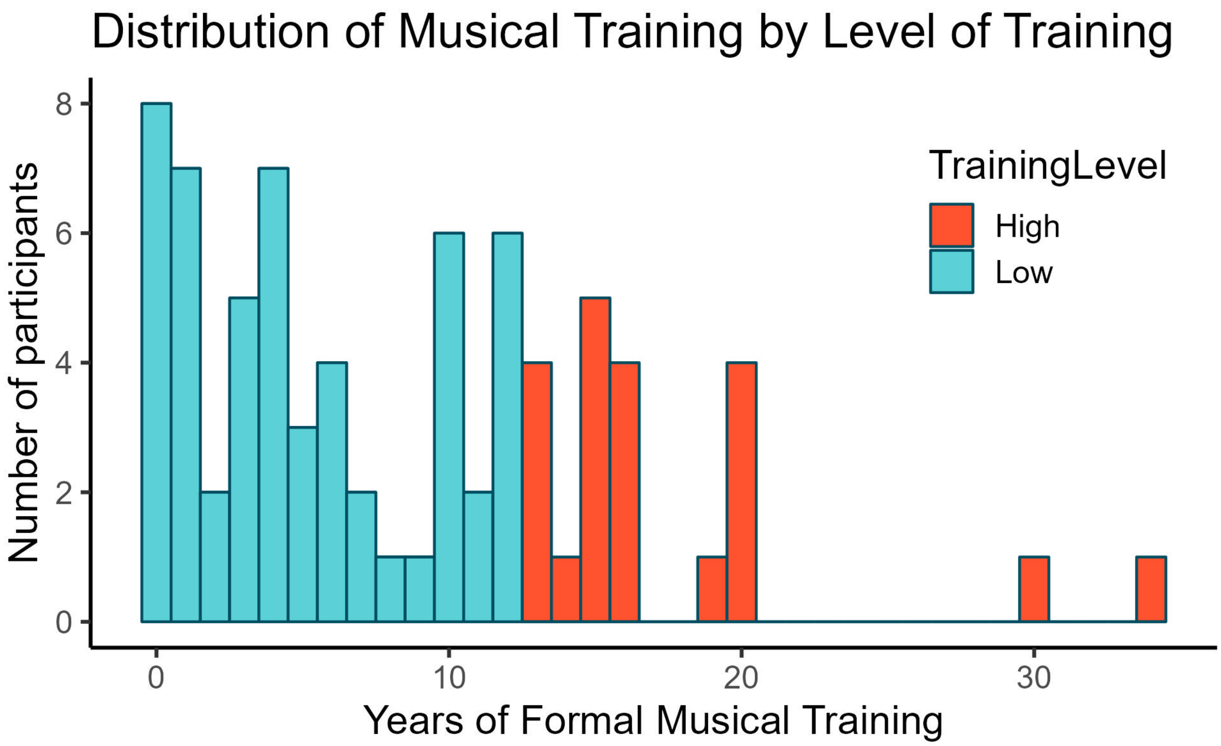

Supplementary Figure 3. Histogram of musician participants by Years of Formal Musical Training with High and Low Training groups highlighted.

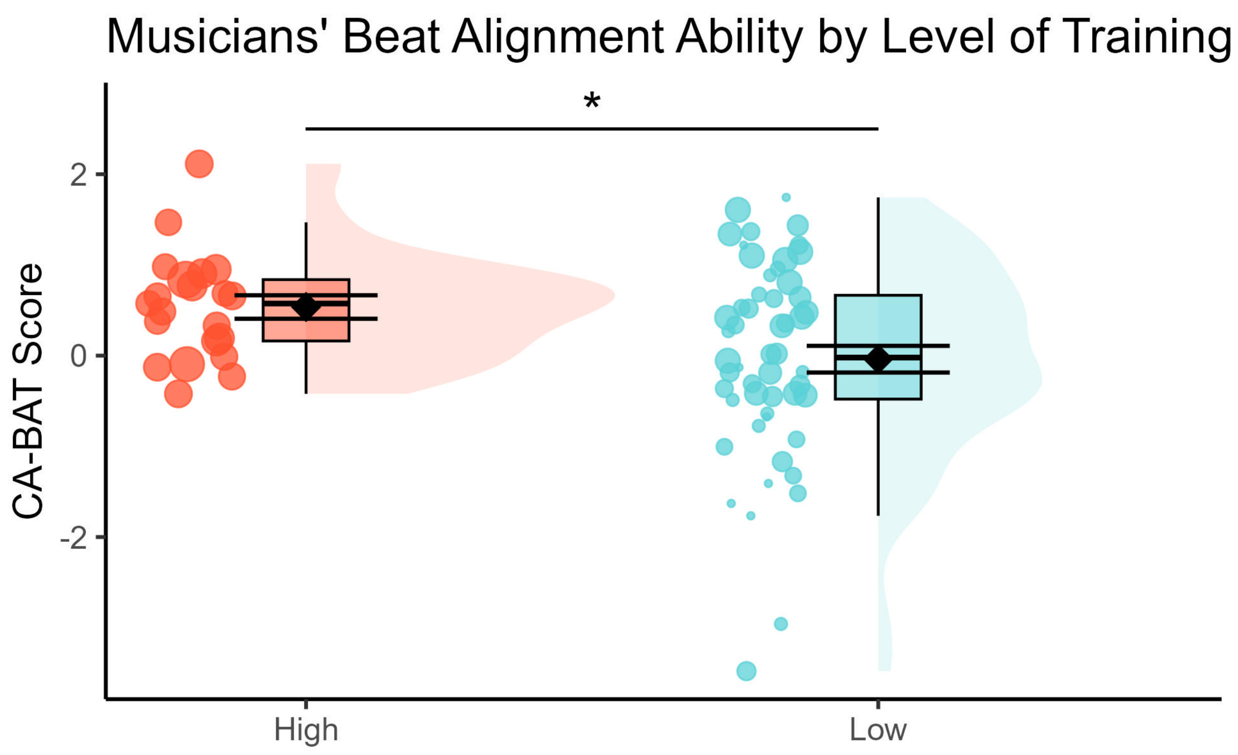

Supplementary Figure 4. CA-BAT scores by High Training and Low Training groups. A small difference was observed between groups as revealed by both the generalized linear model and a Kruskal-Wallis test. Dots are individual subject scores and are scaled in size relative to years of formal musical training while large diamonds are group averages. Error bars represent standard errors of the mean. The boxplots' thick black lines correspond to the group medians, hinges to the first and third quartiles, and whiskers to the most extreme value no further than 1.5 times the interquartile range. The asterisk depicts statistical significance at $p<0.05$.

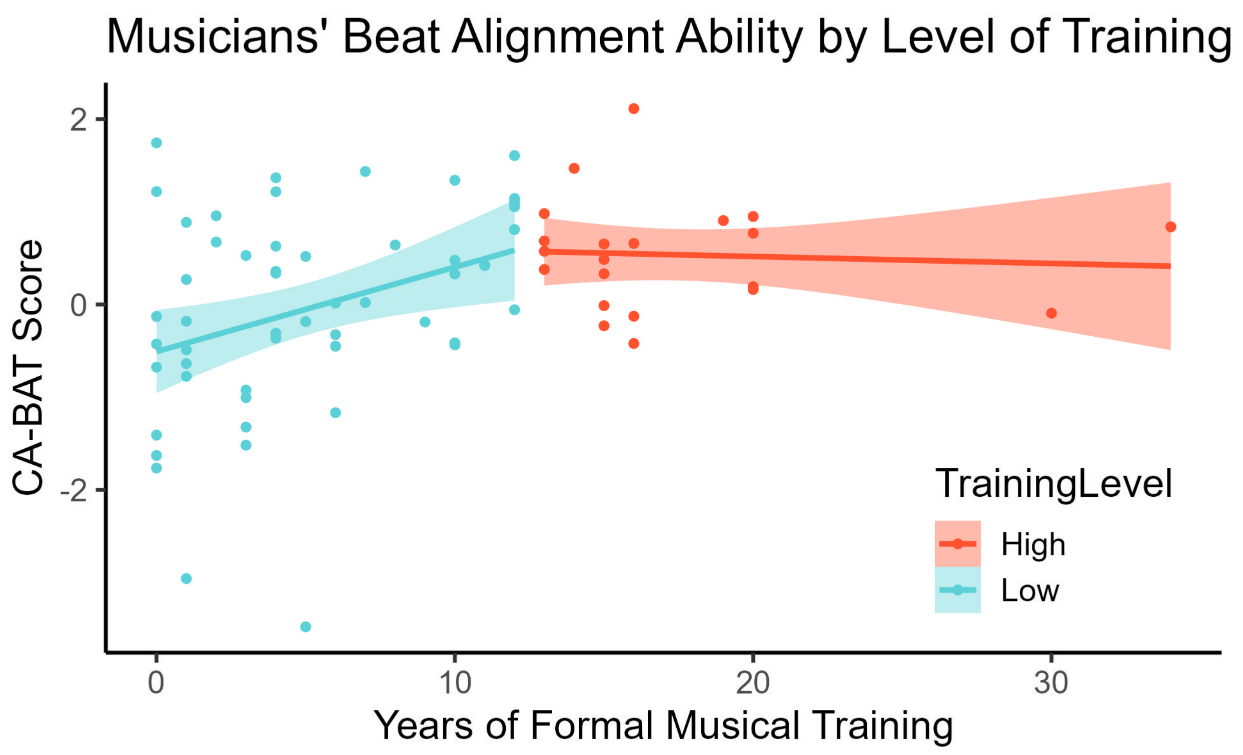

Supplementary Figure 5. Individual CA-BAT scores plotted with regression lines for both High and Low Training groups. There was a positive effect of Years of Formal Musical Training for the Low Training group but no effect for the High Training group.
